# Comparison of methods to quantify macular and peripapillary vessel density in optical coherence tomography angiography

**DOI:** 10.1371/journal.pone.0205773

**Published:** 2018-10-18

**Authors:** Alessandro Rabiolo, Francesco Gelormini, Riccardo Sacconi, Maria Vittoria Cicinelli, Giacinto Triolo, Paolo Bettin, Kouros Nouri-Mahdavi, Francesco Bandello, Giuseppe Querques

**Affiliations:** 1 Department of Ophthalmology, University Vita-Salute, IRCCS San Raffaele, Milan, Italy; 2 Stein Eye Institute, David Geffen School of Medicine, University of California Los Angeles, Los Angeles, California, United States of America; 3 Eye Clinic, Department of Neurological and Movement Sciences, University of Verona, Verona, Italy; Singapore National Eye Centre, SINGAPORE

## Abstract

**Purpose:**

To compare macular and peripapillary vessel density values calculated on optical coherence tomography angiography (OCT-A) images with different algorithms, elaborate conversion formula, and compare the ability to discriminate healthy from affected eyes.

**Methods:**

Cross-sectional study of healthy subjects, patients with diabetic retinopathy, and glaucoma patients (44 eyes in each group). Vessel density in the macular superficial capillary plexus (SCP), deep capillary plexus (DCP), and the peripapillary radial capillary plexus (RCP) were calculated with seven previously published algorithms. Systemic differences, diagnostic properties, reliability, and agreement of the methods were investigated.

**Results:**

Healthy eyes exhibited higher vessel density values in all plexuses compared to diseased eyes regardless of the algorithm used (p<0.01). The estimated vessel densities were significantly different at all the plexuses (p<0.0001) as a function of method used. Inter-method reliability and agreement was mostly poor to moderate. A conversion formula was available for every method, except for the conversion between multilevel and fixed at the DCP. Substantial systemic, non-constant biases were evident between many algorithms. No algorithm outperformed the others for discrimination of patients from healthy subjects in all the retinal plexuses, but the best performing algorithm varied with the selected plexus.

**Conclusions:**

Absolute vessel density values calculated with different algorithms are not directly interchangeable. Differences between healthy and affected eyes could be appreciated with all methods with different discriminatory abilities as a function of the plexus analyzed. Longitudinal monitoring of vessel density should be performed with the same algorithm. Studies adopting vessel density as an outcome measure should not rely on external normative databases.

## Introduction

Optical coherence tomography angiography (OCT-A) is a recent imaging modality that allows non-invasive, rapid, depth-resolved visualization of all the chorioretinal vascular layers. [1, 2] OCT-A devices use different algorithms, all of which are based on the assumption that erythrocytes in the blood vessels are the only moving structures within sequentially acquired B-scans and that they act as a natural motion contrast. Since its recent introduction, OCT-A has gained increased popularity and has been applied to a broad spectrum of disease. [3] En face OCT angiograms can be subjectively evaluated for presence of abnormalities, or can be further post-processed to obtain quantitative, objective metrics. Vessel density, defined as the percentage of the angiocube occupied by retinal vessels, has gained increasing popularity, and represents a promising imaging endpoint for future clinical trials. Its reliability, however, needs to be fully validated. [4] Previous studies demonstrated good intra- and inter-operator repeatability of vessel density for images acquired in the same location, with the same angiocube size, machine, and quantification algorithm. [5, 6] Vessel density results, however, can significantly differ among various devices and depend on angiocube size, scan location, signal strength. [5, 7–9] The reported thresholding algorithms employed to binarize OCT-A angiograms and calculate vessel density, are highly heterogeneous. Some instruments use their own proprietary software, these include Cirrus (AngioPlex software, Carl Zeiss Meditec, Inc., Dublin, CA, USA), [5, 10] AngioVue software (Optovue, Inc., Fremont, CA, USA), [11, 12] and RS -3000 Advance (Nidek, Gamagori, Japan). [6] In the majority of the studies, however, images are exported and post-processed with a variety of different thresholding methods, including fixed cutoffs, [13–15] dynamic cutoff (e.g., mean, [9, 16, 17] ImageJ [National Institutes of Health, Bethesda, MD] default algorithm, [18] Otsu’s algorithm), [19, 20] prototype software, [21, 22] and more complex methods combining preprocessing filters and multilevel thresholds strategies. [23–26] It is still uncertain whether different algorithms lead to the same or, at least, similar results and findings from many studies could have been influenced by the algorithm utilized.

The aims of this study were to compare macular and peripapillary vessel density values calculated with seven different binarization strategies, to calculate conversion formulas between the algorithms, and to compare their diagnostic performance in differentiating healthy subjects from patients affected by diabetic retinopathy (DR) and glaucoma.

## Material and methods

### Study design and patients

One hundred thirty-two eyes of 84 subjects that had OCTA at the Glaucoma Unit, and Medical Retina and Imaging Unit of the Department of Ophthalmology, University Vita-Salute, San Raffaele Hospital, Milan, Italy, were retrospectively evaluated. The study was approved by the San Raffaele Hospital scientific committee, and it adheres to the recommendations of the Declaration of Helsinki. Informed written consent was obtained from all the subjects included. Electronic clinical records, SD-OCT (Cirrus HD-OCT 5000; Carl Zeiss Meditec, Inc.), and swept source (SS)-OCT-A (PLEX Elite 9000, Carl Zeiss Meditec, Inc., Dublin, CA, USA) images from healthy subjects, patients with DR, and patients with glaucoma were reviewed. General inclusion criteria were age ≥18 years old, refractive error between -6 and +3 diopters, and availability of structural OCT and 6x6 mm OCT-A scans with a signal strength ≥ 7. General exclusion criteria included previous ocular surgery other than uncomplicated cataract extraction and intraocular lens implantation performed >6 months before enrollment, and artifacts on OCT-A images. Inclusion criteria specific to the DR group were: type 1 or 2 diabetes, and presence of diabetic retinopathy. Inclusion criteria specific to the glaucoma group were: (i) history of primary open-angle glaucoma; (ii) documented glaucoma damage at the optic disc; (iii) repeatable glaucomatous perimetric damage, defined as a glaucoma hemifield test (GHT) result outside normal limits, and a pattern standard deviation (PSD) with p value <5% normal limits; or (iv) presence of a cluster of ≥3 adjacent points on the pattern deviation plot with a probability of <5%, including at least 1 point having a probability <1% on at least two consecutive standard achromatic visual fields; (v) peripapillary retinal nerve fiber layer (pRNFL) thickness with p <5% in at least one quadrant. Presence of diseases other than DR and glaucoma, respectively, in the DR and glaucoma groups was also an exclusion criterion. Healthy subjects met the following inclusion criteria: (i) no history or evidence of any posterior segment disease; (ii) normal-appearing optic disc and retina on dilated fundus examination; (iii) normal foveal profile on the structural macular SD-OCT scan; (iv) average and quadrant pRNFL thickness within 99% confidence limits; and (v) at least one reliable normal visual field, defined as PSD within 95% confidence limits of the normative database, and a GHT results within normal limits.

### Structural SD-OCT measurements

The structural SD-OCT images consisted of the Optic Disc Cube 200x200, and Macular Cube 512x128 patterns. Control subjects and glaucoma patients had both scans, whereas diabetic patients had only the macular scan. The manufacturer’s software was used to calculate the average pRNFL thickness and central macular thickness (CMT) values on the peripapillary and macular scans, respectively.

### SS-OCTA device and scanning protocol

The SS-OCTA device (PLEX Elite 9000, Carl Zeiss Meditec, Inc., Dublin, CA, USA) uses a swept laser source with a central wavelength of 1040–1060 nm (980–1120 nm full bandwidth) and operates at 100,000 A-scans per second. The axial and transverse resolutions of the system are ∼6 μm and ∼20 μm in tissue, respectively. The OMAG algorithm, which is based on variations in both the intensity and phase information between sequential, co-registered B-scans, was used to generate an OCT-A image. [27, 28] The 6x6 mm angiocube consisted of 500x500 A-scans. En face images consist of a 1024x1024-pixel array with 5.9 μm spacing between pixels. For DR eyes, all scans were centered on the fovea and automated segmentation of the layers carried out to define superficial (SCP) and deep (DCP) capillary plexuses were reviewed. The device’s projections removal algorithm was applied to DCP images. For the glaucoma group, scans were centered on the optic nerve; the segmentation algorithm defined the area between the inner limiting membrane and the outer boundary of the RNFL to isolate the peripapillary radial capillary plexus (RCP). Healthy subjects had both macular and peripapillary OCT-A angiograms. Anonymized raw files were downloaded from the Zeiss PLEX Elite 9000 instruments and uploaded in the Advanced Retina Imaging (ARI) network portal. En face angiograms were then exported in PNG (Portable Network Graphics) format.

### Quantitative analysis of OCT-A images

**Fig 1:**
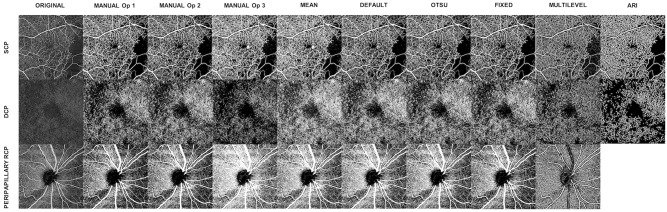
Example of en face angiograms and their binarization with the tested algorithms. Columns and rows show methods and segmented plexuses, respectively.

Seven different threshold strategies were used to binarize en face angiograms and calculate vessel density. The algorithms included the Macular Density algorithm v 0.6.1 developed by Zeiss, a manual thresholding technique, three ImageJ autothresholding algorithm (i.e., mean, default, Otsu), a semiautomatic method using a fixed threshold, and a method combining preprocessing filters with multilevel threshold strategies. Besides the analysis performed with the Zeiss macular density algorithm, all the other ones were carried out using the ImageJ software v 1.51 (National Institutes of Health, Bethesda, MD). At the end of the binarization process, the ratio between the number of white pixels (i.e., vessels) and the number of total pixels was calculated to obtain the vessel density. Fig 1 illustrates an example of binarization outcomes with the employed methods.

#### ARI Zeiss macular density algorithm v 0.6.1

This is a prototype, proprietary algorithm that allows quantification of vascular density both at the SCP and DCP in the macular area. Two indices are generated: perfusion density and vessel density. The former is generated through image binarization, while the latter relies on a skeletonization process. In this manuscript, we included only the first index in our analysis since it is the one based on thresholding, as the other algorithms tested. Since the algorithm was developed for quantification of macular perfusion parameters, it was not used for measuring the peripapillary vasculature. Macular density algorithm was run directly in the ARI portal.

#### Manual thresholding

Three independent operators (AR, RS, FG) independently binarized all the images using a manual, semiautomatic method. Each image was opened twice in the ImageJ software: one was not processed and used as reference, the other was binarized using a manual threshold. Each operator arbitrary chose a cutoff for each image to obtain the best correspondence with the one used as reference.

#### Mean, default, Otsu autothreshold algorithms

Each image was loaded in the ImageJ software and processed using three different autothreshold algorithms: mean, default, and Otsu. Specification of these algorithms can be found online (https://imagej.net/Auto_Threshold). Briefly, the mean algorithm sets the mean level of grey in the image as cutoff. The default is a special method used by ImageJ software, which is a modification of the IsoData algorithm. The Otsu algorithm performs a two cluster-based binarization applying a threshold, which minimizes and maximizes the intra-class and inter-class variances, respectively.

#### Fixed threshold

In this scenario, the same cutoff was utilized for every image of the same plexus. The median level of gray of macular SCP, macular DCP, and peripapillary RCP of the control group was chosen as threshold value. For the macula centered images, we used “59” and “32” for the SCP and DCP, respectively. For the optic nerve centered images, a value of “74” was used as cutoff.

#### Preprocessing filtering with multilevel thresholds strategies

The method used was previously described. [23] Briefly, images were pre-processed using a top-white filter with a window size of 12 pixels. Then, the image was duplicated. One copy underwent binarization through the median auto local threshold; the other one was processed with a Hessian filter and binarized using a Huang autothreshold. The two binarized images were compared and only pixels positive with both methods were counted as vessels.

### Statistical analysis

Data were tested for normality with the Pearson-D’Agostino test. Differences for demographic and main clinical data across groups were evaluated with analysis of variance (ANOVA) and Kruskal-Wallis test, respectively for parametric and non-parametric variables, and Dunnet and Dunn tests were used as post-hoc tests to compare patients with glaucoma and DR groups to healthy subjects for parametric and non-parametric variables, respectively. Differences for categorical variables were assessed with the chi-square test. Differences between glaucoma patients and healthy subjects for VCDR and pRNFL were evaluated with the t-test and the Mann-Whitney test, respectively.

Differences among the algorithms for vessel density values were evaluated with the repeated measures ANOVA or Friedman test for parametric and nonparametric variables, respectively. Pairwise comparisons were investigated with the Tukey test or Dunn’s multiple comparisons as post-hoc tests for parametric and nonparametric data, respectively. Differences in vessel density between healthy and affected eyes at each plexus and with each method were investigated with a linear mixed effect model with presence of disease and age as fixed factors, and fellow eye as random factor.

Inter-algorithm reliability and inter-operator reliability for manual threshold selection were evaluated with the intraclass correlation coefficient (ICC) and the 95% confidence interval (CI) based on a single rating, concordance, 2-way mixed effect model. Values of ICC between 0 and 0.5 indicate poor reliability, moderate reliability between 0.5 and 0.75, good reliability between 0.75 and 0.9, and excellent reliability above 0.9. [29] We used Bland-Altman analysis to evaluate inter-algorithm agreement and limits of agreement (LOA) were set at 1.96 standard deviations (SDs) as this gives the 95% CI.

A structural equation model was used to generate calibration equations to quantify systemic bias between each pair of algorithms, and to elaborate conversion formulas from one method to another.

Ability to discriminate affected eyes from healthy eyes at each plexus was evaluated with receiver operating characteristic (ROC) curves. ROC curves were estimated on a generalized linear model in order to adjust for age, and eyes of the same patients were considered as clustered to account for correlations between eyes. [30] Area under the curve (AUROC) values and 95% confidence intervals were calculated. AUROCs of 0.5 and 1 represent lack of and perfect discrimination, respectively. Pairs of ROC curves were compared with the DeLong’s test.

All statistical analyses were performed with R software (R Foundation for Statistical Computing, Vienna, Austria, https://www.r-project.org), GraphPad Prism software 6.0 (GraphPad Software, Inc., San Diego, CA, USA), and SPSS software 21 (SPSS Inc., Chicago, IL, USA). A p-value <0.05, after adjustments with the Benjamini-Hochberg test, was considered significant.

## Results

Table 1 summarizes the demographic and main clinical data of the cohort of patients. Patients in the glaucoma groups were significantly older than those in control group (p = 0.008), and they had increased VCDR (p <0.0001) and reduced pRNFL thickness (<0.0001). Patients with DR had significantly greater CMT compared with controls (p = 0.001), and 18 eyes (40.9%) featured diabetic macular edema. In the DR group, 28 and 3 patients suffered from type 1 and 2 diabetes, respectively, with a mean hemoglobin A1c of 6.9 ± 1.1%. Severity of DR was mild in 1 eye, moderate in 6 eyes, severe in 8 eyes, proliferative in 28 eyes, and unknown in 1 eye.

**Table 1:** Demographic and main clinical data of study population.

Parameters	Overall	Controls	DR	p-value	Glaucoma	p-value
No. Patients / Eyes	132 / 84	27 / 44	31 / 44	n/a	26 / 44	n/a
Age years, mean ± SD	54.2 ± 16.2	47.7 ± 17.9	54.6 ± 15.4	0.17	60.5 ± 13.1	**0.008**
Race, caucasian	84	27	31	n/a	26	n/a
Sex, male / female	37/ 47	15 / 12	11 / 20	0.79	11 / 15	0.63
Eye, right / left	63 / 69	22 / 22	20 / 24	0.91	21 / 23	0.98
CMT, μm, mean ± SD	282.9 ± 57.8	265.8 ± 21.8	310.0 ± 82.9	**0.0014**	268.2 ± 29.0	0.99
VCDR, mean ± SD	0.61 ± 0.17*	0.52 ± 0.17	n/a	n/a	0.70 ± 0.13	**< 0.0001**
pRNFL, μm, mean ± SD	81.6 ± 13.8*	88.8 ± 10.9	n/a	n/a	74.5 ± 12.6	**< 0.0001**

*This value does not include eyes belonging to DR group. BCVA: best corrected visual acuity; CMT: central macular thickness; DR: diabetic retinopathy; SD: standard deviation; CMT: central macular thickness; VCDR: vertical cup-to-disc ratio; pRNFL: peripapillary retinal nerve fiber layer.

**Fig 2:**
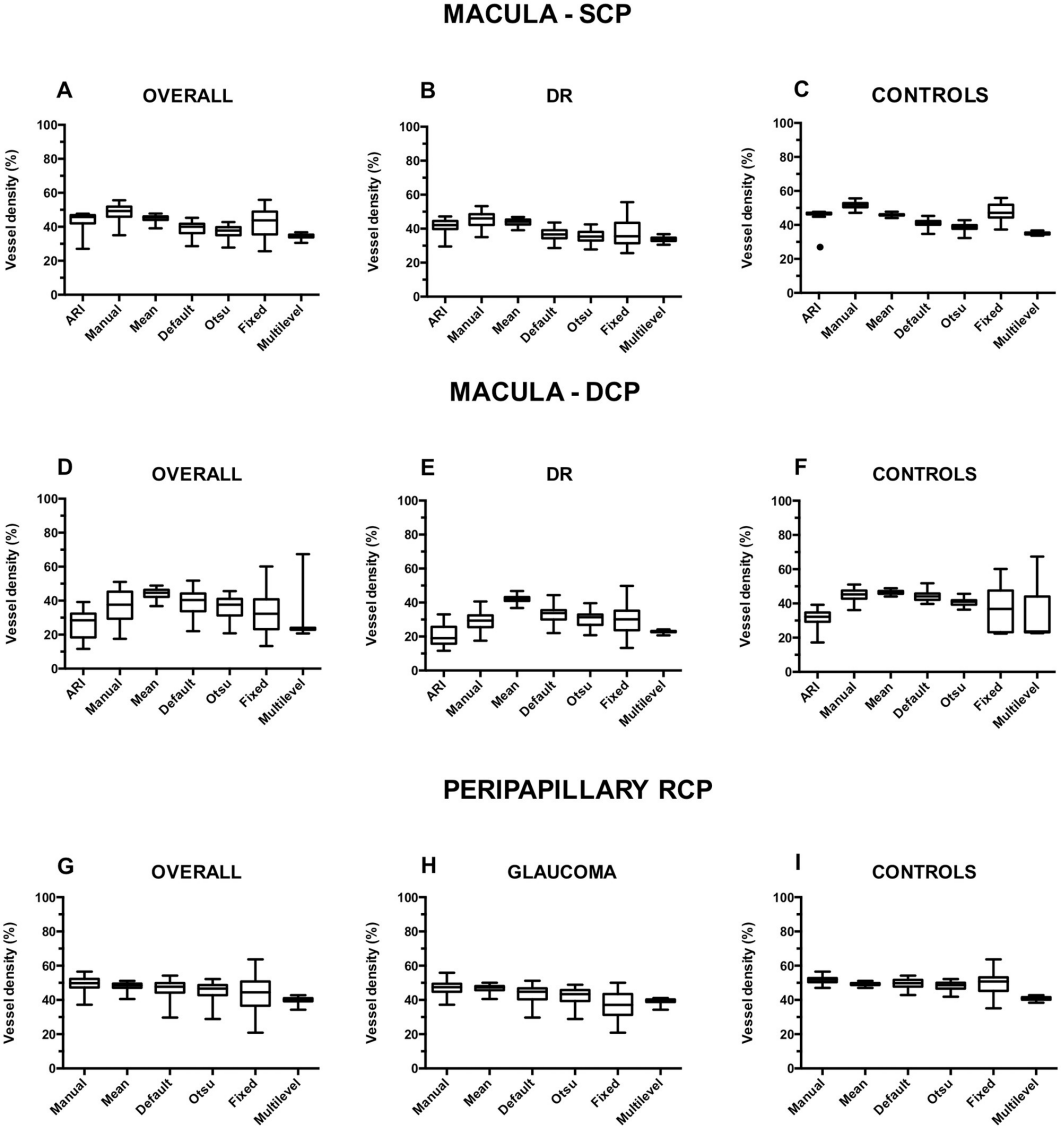
Vessel density values calculated with the tested methods. Vessel density values calculated with the tested methods for the entire cohort, diabetic retinopathy (DR) and glaucoma patients for the macular superficial capillary plexus (SCP) **(A,B,C)**, deep capillary plexus (DCP) **(D,E,F)**, and peripapillary radial capillary plexus (RCP) **(G,H,I)**. Processing methods differences were significant at p < 0.0001 at all levels. SCP: superficial capillary plexus; DCP: deep capillary plexus; RCP: radial capillary plexus; DR: diabetic retinopathy.

Fig 2 and Table 2 show the macular SCP, DCP and peripapillary RCP vessel density values estimated with the different methods. Healthy eyes exhibited higher vessel density values at all plexuses compared with eyes with disease regardless the algorithm used (p < 0.01). No significant difference between diabetic eyes with and without DME estimated with every method at both SCP and DCP was found (p-value > 0.31). Vessel density values were significantly different among the different methods at all the plexuses (p < 0.0001). All the pairwise comparisons for the macular SCP (Table 3) were significant (p < 0.05), except for the pairs ARI and mean (p = 0.99), ARI and fixed (p = 0.70), mean and fixed (p = 0.10), and Otsu and multilevel (p = 0.20). At the macular DCP level (Table 4), all the pairwise comparisons were significant (p< 0.01), except for the pairs ARI and multilevel (p = 0.99), manual and default (0.99), manual and Otsu (p = 0.13), manual and fixed (p = 0.49), Otsu and fixed (0.99). At the peripapillary RCP level (Table 5), all pairs had significantly different vessel density values (p < 0.001), except for manual and mean (p = 0.08), mean and default (p = 0.99), and Otsu and multilevel (p = 0.99).

**Table 2:** Vessel density values estimated with the different methods.

	Macula—superficial capillary plexus			Macula—deep capillary plexus			ONH—radial peripapillary capillary plexus		
	Overall	DR patients	Healthy subjects	Overall	DR patients	Healthy subjects	Overall	Glaucoma patients	Healthy subjects
**ARI**	43.9 ± 4.1	41.6 ± 3.7	46.2 ± 3.0	26.2 ± 7.6	20.8 ± 6.0	31.7 ± 4.5	n/a	n/a	n/a
**Manual**	48.6 ± 4.4	45.5 ± 4.1	51.6 ± 1.9	37.1 ± 9.0	29.4 ± 5.2	44.9 ± 3.7	49.6 ± 3.7	47.3 ± 3.5	51.8 ± 2.2
**Mean**	44.9 ± 1.7	43.9 ± 1.9	45.9 ± 0.8	44.3 ± 2.7	42.2 ± 2.0	46.4 ± 1.2	48.0 ± 2.1	46.7 ± 2.2	49.3 ± 0.9
**Default**	39.1 ± 3.6	37.1 ± 3.6	41.1 ± 2.2	38.6 ± 6.6	33.0 ± 4.3	44.2 ± 2.5	46.4 ± 5.2	43.4 ± 5.4	49.5 ± 2.5
**Otsu**	37.3 ± 3.3	35.8 ± 3.5	38.8 ± 2.2	35.5 ± 6.2	30.3 ± 4.3	40.7 ± 2.0	45.0 ± 5.1	41.9 ± 5.2	48.1 ± 2.5
**Fixed**	42.5 ± 8.3	37.5 ± 8.0	47.5 ± 4.9	33.3 ± 10.9	30.3 ± 8.1	36.3 ± 12.6	43.4 ± 9.2	37.1 ± 7.2	49.7 ± 6.1
**Multilevel**	34.5 ± 1.3	33.9 ± 1.5	35.1 ± 0.7	27.4 ± 9.6	22.9 ± 0.7	31.9 ± 12.1	40.0 ± 1.5	39.3 ± 1.5	40.8 ± 1.0

Data are presented as mean ± standard deviation. DR: diabetic retinopathy; ONH: optic nerve head.

**Table 3:** Inter-algorithm agreement, reliability, and pairwise comparison in the superficial capillary plexus for the entire cohort of patients.

Algorithm comparison		Agreement (Bland Altman Analysis)			Reliability	Pairwise comparison
*Algorithm 1*	*Algorithm 2*	*MD*	*LoA*	*Range*	*ICC (95% CI)*	*P Value*
ARI	Manual	-4.6	-11.4/2.1	13.5	0.674 (0.542–0.774)	**< 0.0001**
ARI	Mean	-1.0	-6.8/4.8	11.6	0.556 (0.393–0.685)	0.99
ARI	Default	4.8	0.1/9.6	9.5	**0.800 (0.710–0.864)**	**< 0.0001**
ARI	Otsu	6.7	1.7/11.6	9.9	**0.768 (0.667–0.842)**	**< 0.0001**
ARI	Fixed	1.4	-9.6/12.5	22.1	0.625 (0.479–0.378)	0.70
ARI	Multilevel	9.4	3.1/15.8	12.7	0.428 (0.241–0.584)	**< 0.0001**
Manual	Mean	3.7	-2.6/9.9	12.5	0.552 (0.389–0.682)	**0.0012**
Manual	Default	9.5	4.0/15.0	11.0	**0.756 (0.650–0.833)**	**< 0.0001**
Manual	Otsu	11.3	5.0/17.6	12.6	0.654 (0.516–0.759)	**< 0.0001**
Manual	Fixed	6.1	-5.3/17.4	22.7	0.620 (0.472–0.733)	**< 0.0001**
Manual	Multilevel	14.1	7.0/21.1	14.1	0.393 (0.201–0.556)	**< 0.0001**
Mean	Default	5.8	2.0/9.7	7.9	**0.760 (0.655–0.836)**	**< 0.0001**
Mean	Otsu	7.6	4.2/11.1	6.9	**0.775 (0.676–0.847)**	**< 0.0001**
Mean	Fixed	2.4	-10.9/15.8	26.7	0.351 (0.154–0.521)	0.10
Mean	Multilevel	10.4	8.6/12.2	3.6	**0.830 (0.751–0.885)**	**< 0.0001**
Default	Otsu	1.8	0.3/3.3	3.0	**0.976 (0.963–0.984)**	**0.0109**
Default	Fixed	-3.4	-13.7/6.9	20.6	0.663 (0.527–0.765)	**0.0220**
Default	Multilevel	4.6	-0.4/9.6	10.0	0.562 (0.400–0.690)	**< 0.0001**
Otsu	Fixed	-5.2	-16.2/5.8	22.0	0.602 (0.449–0.720)	**< 0.0001**
Otsu	Multilevel	2.8	-1.7/7.2	8.9	0.585 (0.429–0.708)	0.20
Fixed	Multilevel	8.0	-6.1/22.2	28.3	0.257 (0.051–0.441)	**< 0.0001**

MD: mean difference; LoA: limits of agreement; ICC: intraclass correlation coefficient; CI: confidence interval

**Table 4:** Inter-algorithm agreement, reliability, and pairwise comparison in the deep capillary plexus for the entire cohort of patients.

Algorithm comparison		Agreement (Bland Altman Analysis)			Reliability	Pairwise comparison
*Algorithm 1*	*Algorithm 2*	*MD*	*LoA*	*Range*	*ICC (95% CI)*	*P Value*
ARI	Manual	-10.9	-23.8/1.9	25.7	0.689 (0.561–0.784)	**< 0.0001**
ARI	Mean	-18.0	-29.1/-6.9	22.2	0.502 (0.328–0.643)	**< 0.0001**
ARI	Default	-12.4	-21.1/-3.6	17.5	**0.804 (0.715–0.867)**	**< 0.0001**
ARI	Otsu	-9.3	-17.9/-0.7	17.2	**0.799 (0.709–0.864)**	**< 0.0001**
ARI	Fixed	-7.1	-24.7/10.6	35.3	0.540 (0.373–0.673)	**< 0.0001**
ARI	Multilevel	-1.2	-19.8/17.5	37.3	0.397 (0.205–0.559)	0.99
Manual	Mean	-7.1	-20.8/6.5	27.3	0.445 (0.260–0.598)	**< 0.0001**
Manual	Default	-1.4	-10.2/7.2	17.4	**0.842 (0.768–0.894)**	0.99
Manual	Otsu	1.6	-7.3/10.5	17.8	**0.827 (0.748–0.883)**	0.13
Manual	Fixed	3.8	-19.8/27.5	47.3	0.274 (0.069–0.456)	0.49
Manual	Multilevel	9.8	-8.0/27.5	35.5	0.525 (0.356–0.611)	**< 0.0001**
Mean	Default	5.7	-2.5/13.8	16.3	0.662 (0.527–0.765)	**0.0021**
Mean	Otsu	8.7	1.4/16.0	14.6	0.693 (0.566–0.788)	**< 0.0001**
Mean	Fixed	11.0	-8.7/30.6	39.3	0.205 (-0.003–0.397)	**< 0.0001**
Mean	Multilevel	16.9	-0.3/34.1	34.4	0.225 (0.017–0.414)	**< 0.0001**
Default	Otsu	3.1	0.4/5.7	5.3	**0.978 (0.966–0.985)**	**< 0.0001**
Default	Fixed	5.3	-14.3/24.9	39.2	0.388 (0.195–0.552)	**0.0008**
Default	Multilevel	11.2	-6.1/28.5	34.6	0.430 (0.243–0.586)	**< 0.0001**
Otsu	Fixed	2.3	-16.9/21.3	38.2	0.398 (0.206–0.560)	0.99
Otsu	Multilevel	8.2	-9.1/25.4	34.5	0.408 (0.219–0.568)	**0.0002**
Fixed	Multilevel	5.9	-27.9/39.8	67.7	-0.409 (-0.569 –-0.219)	**< 0.0001**

MD: mean difference; LoA: limits of agreement; ICC: intraclass correlation coefficient; CI: confidence interval

**Table 5:** Inter-algorithm agreement, reliability, and pairwise comparison in the peripapillary radial capillary plexus for the entire cohort of patients.

Algorithm comparison		Agreement (Bland Altman Analysis)			Reliability	Pairwise comparison
*Algorithm 1*	*Algorithm 2*	*MD*	*LoA*	*Range*	*ICC (95% CI)*	*P Value*
Manual	Mean	1.6	-3.2/6.3	9.5	0.672 (0.539–0.772)	0.08
Manual	Default	3.2	-3.6/9.9	13.5	0.709 (0.588–0.800)	**0.0005**
Manual	Otsu	4.6	-2.0/11.1	13.1	0.718 (0.599–0.806)	**< 0.0001**
Manual	Fixed	6.2	-7.3/19.7	27.0	0.518 (0.347–0.656)	**< 0.0001**
Manual	Multilevel	9.6	3.7/15.4	11.7	0.439 (0.254–0.593)	**< 0.0001**
Mean	Default	1.6	-4.5/7.7	12.2	0.690 (0.563–0.786)	0.99
Mean	Otsu	3.0	-3.0/9.0	12.0	0.693 (0.567–0.788)	**< 0.0001**
Mean	Fixed	4.6	-9.8/19.0	28.8	0.390 (0.198–0.554)	**< 0.0001**
Mean	Multilevel	8.0	5.7/10.3	4.6	**0.789 (0.694–0.856)**	**< 0.0001**
Default	Otsu	1.4	0.3/2.5	2.2	**0.994 (0.991–0.996)**	**< 0.0001**
Default	Fixed	3.0	-7.0/13.1	20.1	**0.765 (0.662–0.840)**	**< 0.0001**
Default	Multilevel	6.4	-1.5/14.3	15.8	0.442 (0.257–0.596)	**< 0.0001**
Otsu	Fixed	1.6	-8.4/11.6	20.0	**0.764 (0.661–0.839)**	0.99
Otsu	Multilevel	5.0	-2.7/12.7	15.4	0.452 (0.269–0.603)	**0.0006**
Fixed	Multilevel	3.4	-12.5/19.2	31.7	0.246 (0.039–0.432)	**< 0.0001**

MD: mean difference; LoA: limits of agreement; ICC: intraclass correlation coefficient; CI: confidence interval

Inter-algorithm ICC values of the SCP, DCP, and RCP are illustrated in Tables 3, 4 and 5, respectively. Default and Otsu algorithms had an excellent reliability at every plexus. Reliability of ARI algorithm was good when compared with default and Otsu methods. At the SCP, a good reliability was observed also for the pairs mean and default, mean and Otsu, and mean and multilevel. At the DCP, the manual algorithm had a good reliability in comparison with both default and Otsu methods. The pairs mean and multilevel, default and fixed, and Otsu and fixed had a good reliability at the peripapillary RCP. All the other pairs exhibited a moderate to poor reliability. Notably, fixed and multilevel had a negative ICC at the DCP since their vessel density values were negatively correlated.

Results of Bland-Altman analysis for the inter-method agreement of the SCP, DCP, and RCP are also displayed in Tables 3, 4 and 5, respectively. Limits of agreement and mean differences were wider in the DCP, indicating a lower level of agreement between methods for this plexus. Otsu and default algorithms exhibited an excellent agreement. On the contrary, the fixed algorithm had a poor agreement with all the other methods.

**Fig 3:**
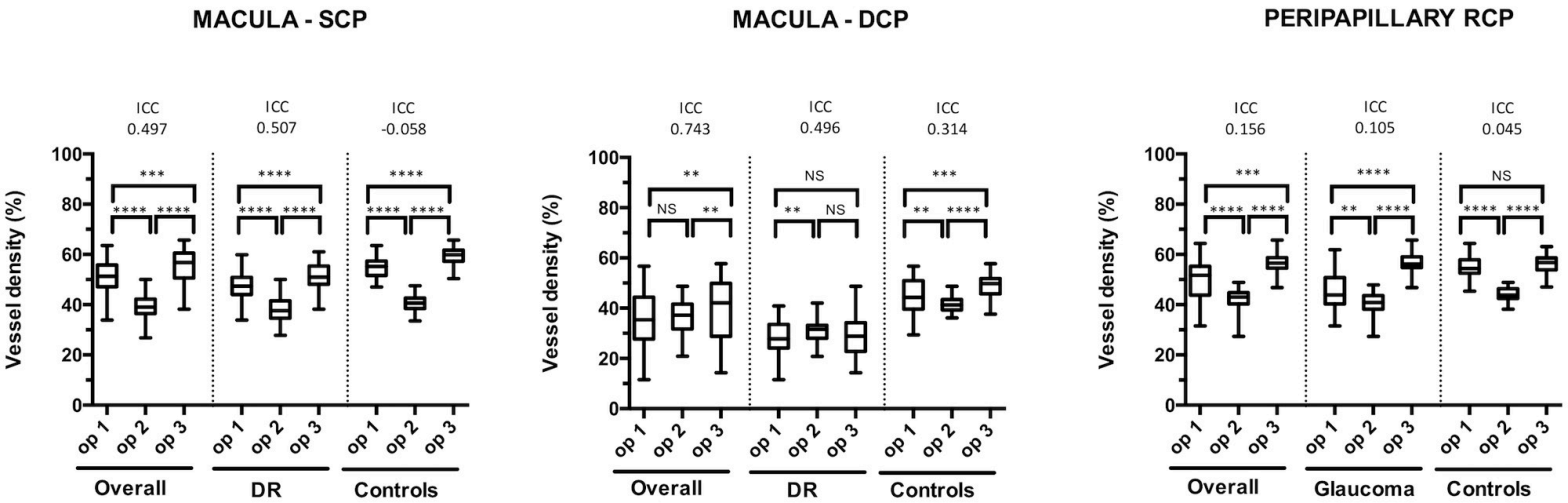
Inter-rater reliability of the manual method for the entire cohort, DR and glaucoma patients. **** significant at p < 0.0001; *** significant at p < 0.001; ** significant at p < 0.01; NS: not significant; DR: diabetic retinopathy; ICC: intraclass correlation coefficient; op: operator; SCP: macular superficial capillary plexus; DCP: macular deep capillary plexus; RCP: peripapillary radial capillary plexus.

As shown in Fig 3, the inter-operator reliability for the manual method was moderate for the DCP overall and in the subset of patients with DR, and poor for all the other groups. Vessel density values were significantly different between each pair of operator, except the comparison between operator 1 and 2 at the DCP for the cohort of all patients, and between operator 3 and both operators 1 and 2 for the DCP of the patients with DR.

**Fig 4:**
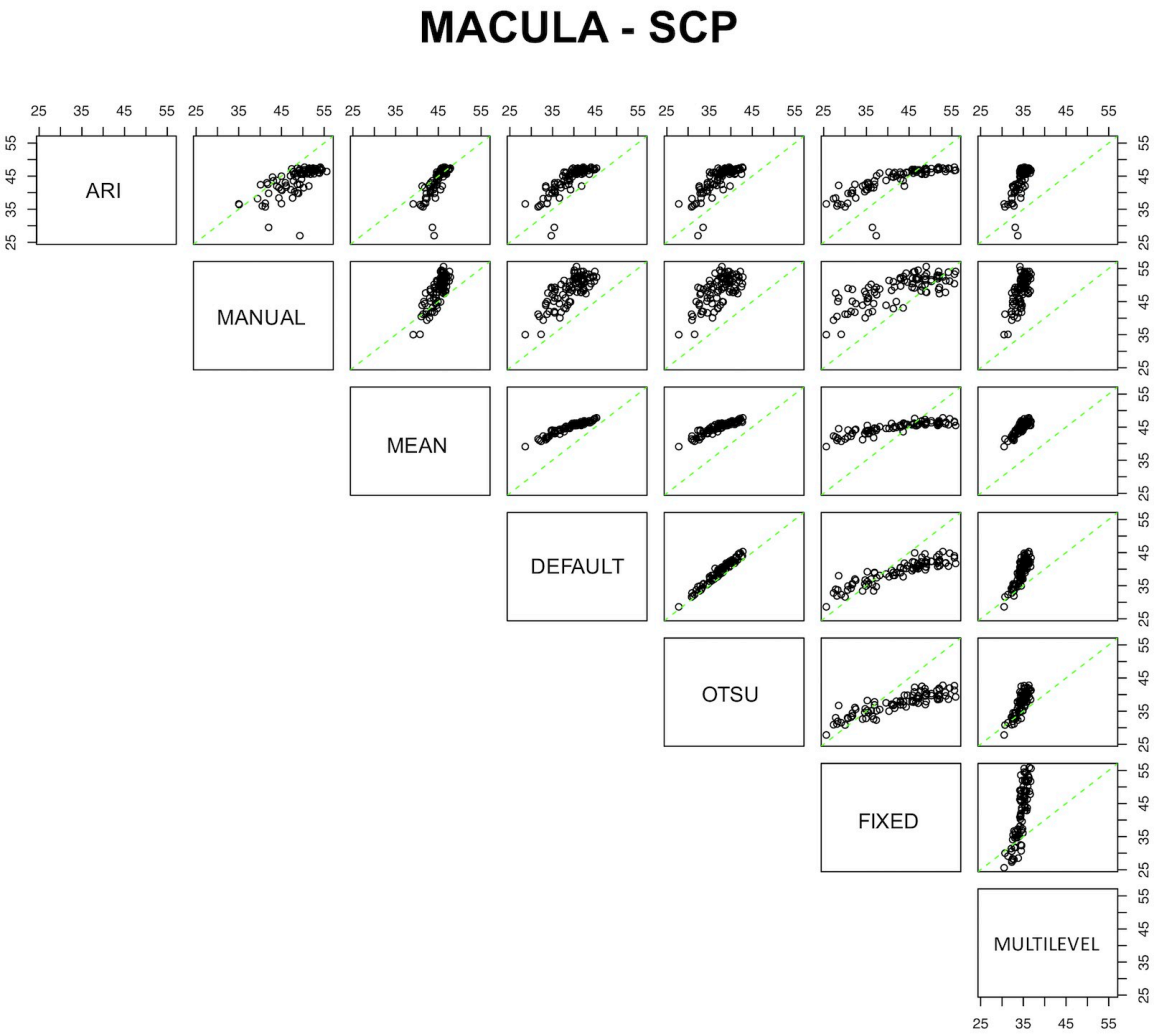
Comparative plots for the seven processing methods for the superficial capillary plexus (SCP). Green lines represent the no-bias line, while black circles demonstrate the true corresponding measurement among coupled devices.

**Fig 5:**
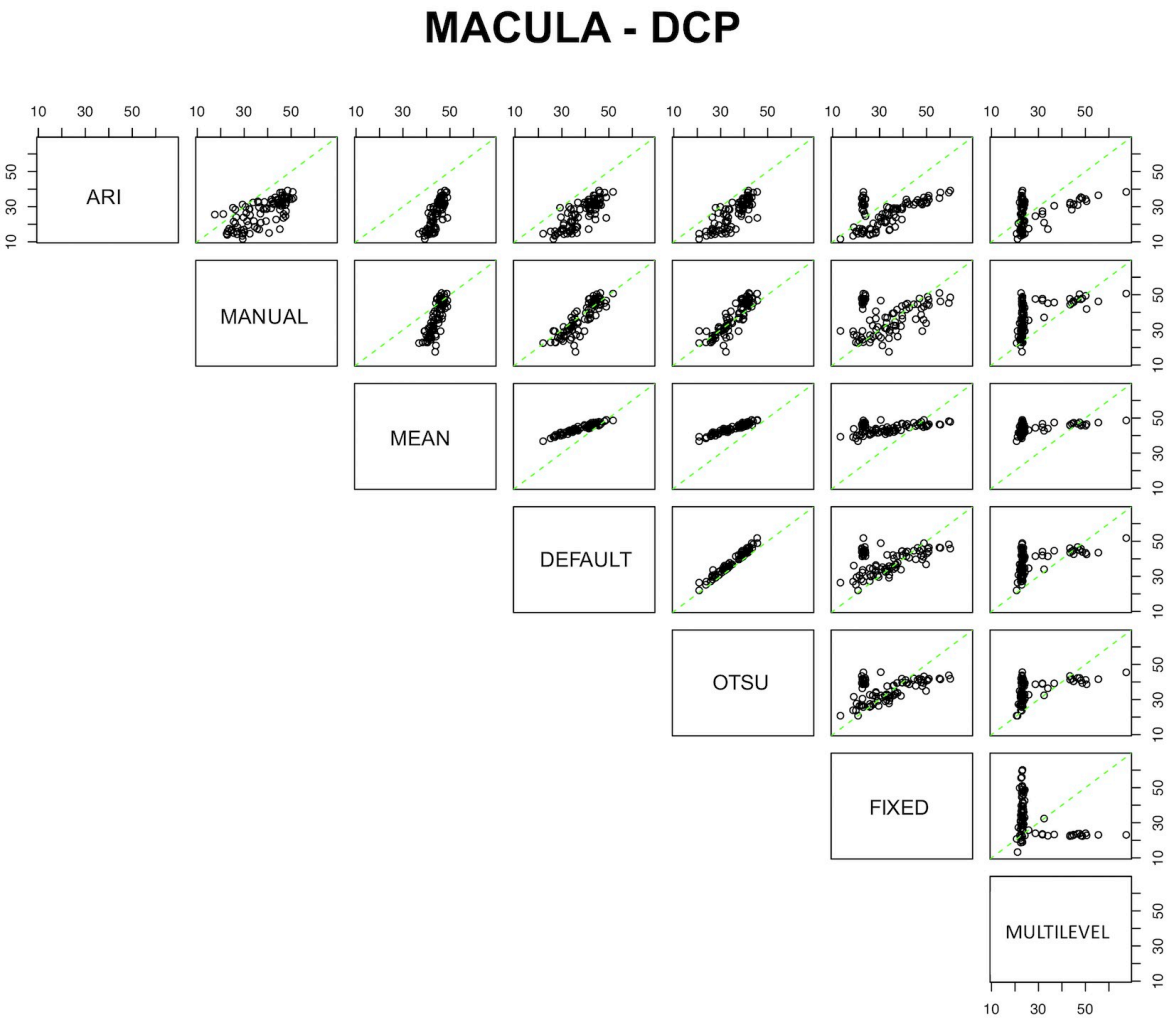
Comparative plots for the seven processing methods for the deep capillary plexus (DCP). Green lines represent the no-bias line, while black circles show the true corresponding measurement among coupled devices.

**Fig 6:**
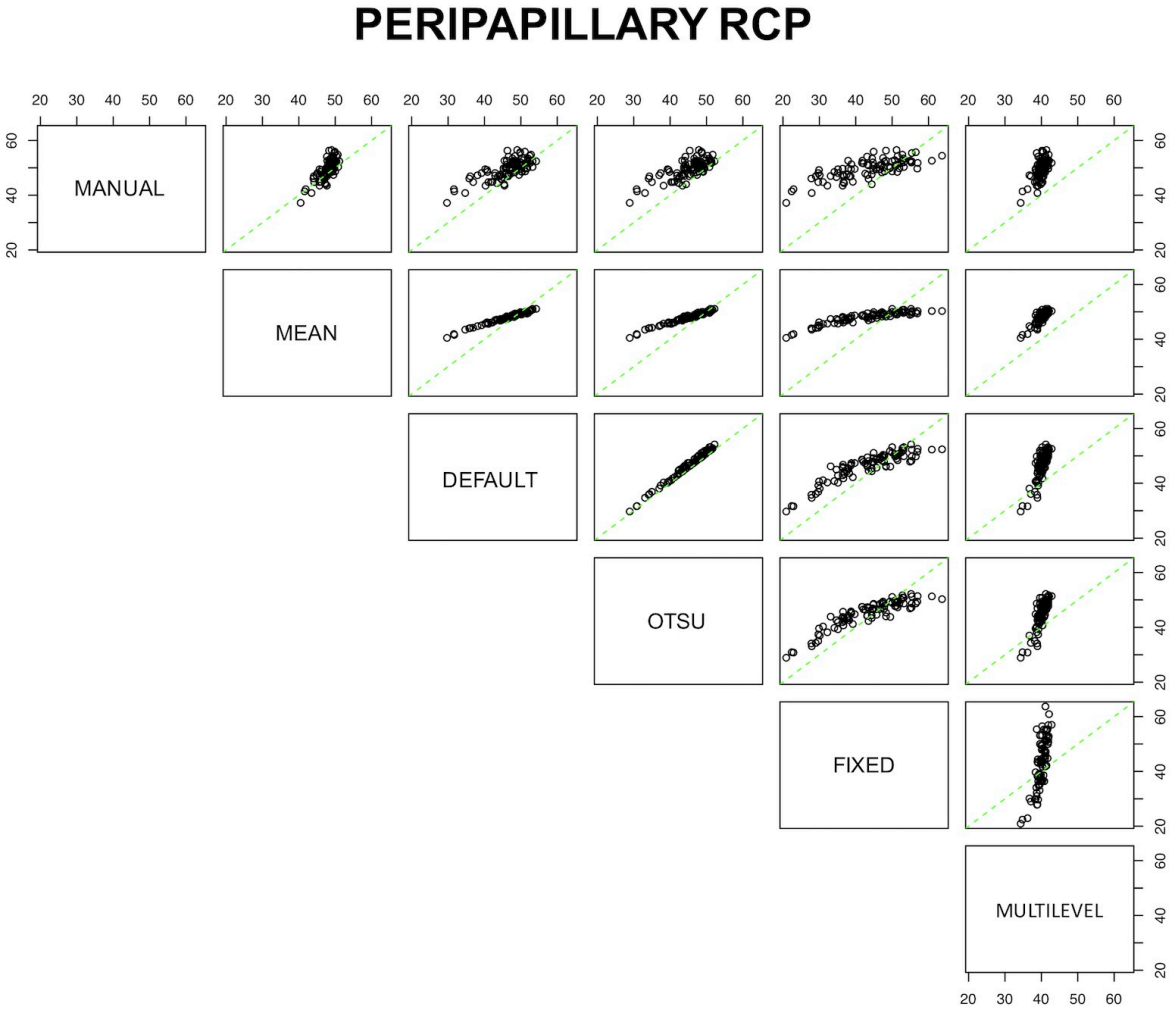
Comparative plots for the seven processing methods for the peripapillary radial capillary plexus (RCP). Green lines represent the no-bias line, while black circles demonstrate the true corresponding measurement among coupled devices.

Table 6 displays the calibration equations for the methods. The conversion formula from one instrument to another was available for all the tested algorithms, except for the conversion from multilevel to fixed (and vice versa) at the DCP since observed values measured by these two algorithms were negatively correlated. Notably, the default and Otsu algorithms at the peripapillary RCP had almost no bias, and a small bias at the other plexuses, meaning that the systematic error between these two algorithms is extremely low. A substantial bias was evident between many algorithms. The comparative plots for the SCP, DCP, and peripapillary RCP are graphically shown in Figs 4, 5 and 6, respectively.

**Table 6:** Calibration equations to convert vessel density value from one algorithm into the equivalent from another algorithm.

Algorithm	Calibration equation		
	Macula—SCP	Macula—DCP	Peripapillary RCP
ARI	-2.86 + 0.96 * Manual	-9.68 + 0.97 * Manual	n/a
	-45.51 + 1.99 * Mean	-93.05 + 2.70 * Mean	n/a
	6.76 + 0.95 * Default	-14.45 + 1.05 * Default	n/a
	3.02 + 1.10 * Otsu	-13.34 + 1.11 * Otsu	n/a
	25.11 + 0.44 * Fixed	-19.04 + 1.36 * Fixed	n/a
	-53.29 + 2.88 * Multilevel	-10.49 + 1.34 * Multilevel	n/a
Manual	2.97 + 1.04 * ARI	10.01 + 1.03 * ARI	n/a
	-44.27 + 2.07 * Mean	-86.21 + 2.79 * Mean	-15.83 + 1.36 * Mean
	9.99 + 0.99 * Default	-4.93 + 1.09 * Default	23.58 + 0.56 * Default
	6.11 + 1.14 * Otsu	-3.78 + 1.15 * Otsu	24.13 + 0.57 * Otsu
	35.46 + 0.22 * Fixed	-9.68 + 1.41 *Fixed	34.94 + 0.34 * Fixed
	-54.42 + 2.99 * Multilevel	-11.66 + 1.77 * Multilevel	-41.82 + 2.28 * Multilevel
Mean	21.42 + 0.48 * ARI	34.53 + 0.37 * ARI	n/a
	21.16 + 0.49 * Manual	31.11 + 0.36 * Manual	11.62 + 0.73 * Manual
	26.25 + 0.48 * Default	29.17 + 0.39 * Default	28.93 + 0.41 * Default
	24.37 + 0.55 * Otsu	29.58 + 0.41 * Otsu	29.3 + 0.42 * Otsu
	35.21 + 0.23 * Fixed	27.46 + 0.51 * Fixed	32.27 + 0.25 * Fixed
	-4.91 + 1.45 * Multilevel	29.44 + 0.54 * Multilevel	-19.08 + 1.68 * Multilevel
Default	-7.10 + 1.05 * ARI	13.71 + 0.95 * ARI	n/a
	-10.12 + 1.01 * Manual	4.52 + 0.92 * Manual	-42.10 + 1.79 * Manual
	-54.97 + 2.09 * Mean	-74.55 + 2.56 * Mean	-70.36 + 2.43 * Mean
	-3.93 + 1.15 * Otsu	1.05 + 1.06 * Otsu	0.98 + 1.01 * Otsu
	19.30 + 0.47 * Fixed	-4.36 + 1.29 * Fixed	20.29 + 0.60 * Fixed
	-65.25 + 3.03 *Multilevel	0.06 + 1.40 * Multilevel	-116.76 + 4.08 *Multilevel
Otsu	-2.75 + 0.91 * ARI	11.98 + 0.90 * ARI	n/a
	-5.36 + 0.88 * Manual	3.29 + 0.87 * Manual	-42.65 + 1.77 * Manual
	-44.23 + 1.82 * Mean	-71.59 + 2.42 * Mean	-70.62 + 2.41 * Mean
	3.41 + 0.87 * Default	-1.00 + 0.95 * Default	-0.97 + 0.99 * Default
	20.13 + 0.40 * Fixed	-5.12 + 1.22 * Fixed	19.12 + 0.60 * Fixed
	-53.14 + 2.62 * Multilevel	-0.22 + 1.30 * Multilevel	-116.57 + 4.04 * Multilevel
Fixed	-56.71 + 2.26 * ARI	14.00 + 0.74 * ARI	n/a
	-63.18 + 2.18 * Manual	6.88 + 0.71 *Manual	-130.58 + 2.96 * Manual
	-159.50 + 4.50 * Mean	-54.42 + 1.98 * Mean	-150.49 + 4.04 * Mean
	-41.45 + 2.15 * Default	3.38 + 0.78 * Default	-33.69 + 1.66 * Default
	-49.89 + 2.48 Otsu	4.19 + 0.82 * Otsu	-32.06 + 1.68 * Otsu
	-181.59 + 6.50 * Multilevel	n/a*	-227.53 + 6.77 * Multilevel
Multilevel	19.22 + 0.35 * ARI	-7.86 + 0.75 * ARI	n/a
	18.22 + 0.34 * Manual	6.58 + 0.56 * Manual	18.31 + 0.44 * Manual
	3.40 + 0.69 * Mean	-54.65 + 1.86 * Mean	11.38 + 0.60 * Mean
	21.57 + 0.33 * Default	-0.04 + 0.71 * Default	28.64 + 0.25 * Default
	20.27 + 0.38 * Otsu	0.17 + 0.77 * Otsu	28.88 + 0.25 * Otsu
	27.95 + 0.15 * Fixed	n/a*	33.61 + 0.15 * Fixed

* Calibration equation not available since fixed and multilevel algorithms were negative correlated at the DCP. SCP: superficial capillary plexus; DCP: deep capillary plexus; RPC: radial peripapillary capillaries.

**Fig 7:**
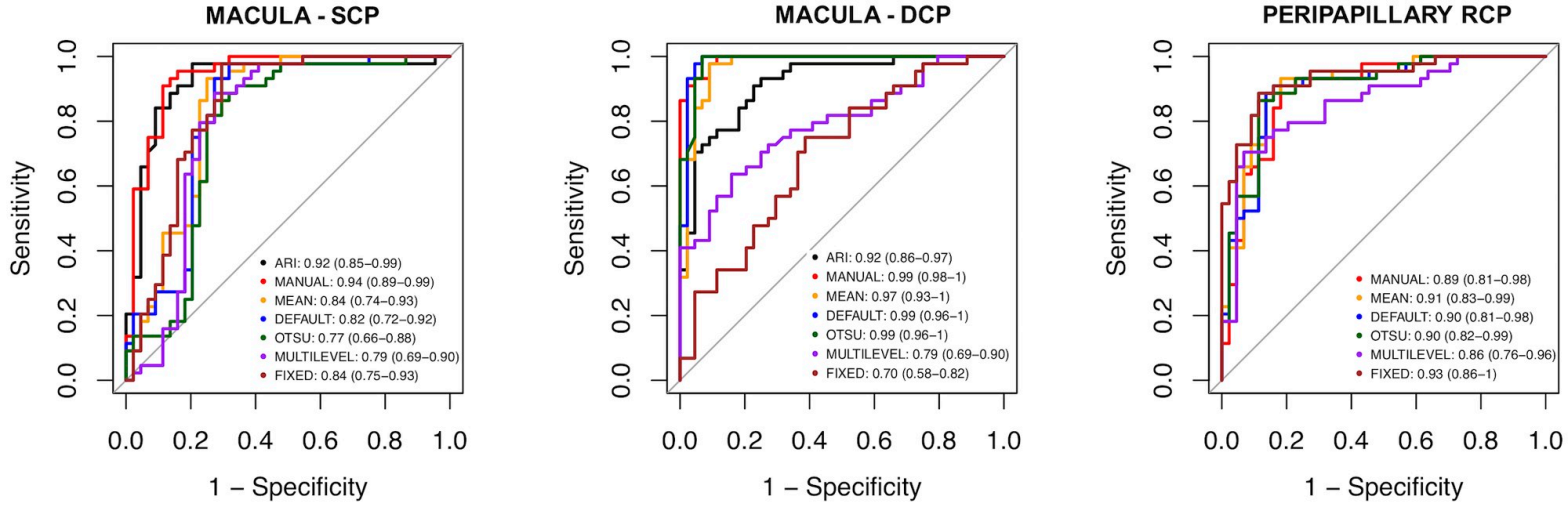
Receiver operating characteristic (ROC) curves for discrimination of diseased from healthy eyes. Legends show area under the ROC curve and 95% confidence interval. SCP: macular superficial capillary plexus; DCP: macular deep capillary plexus; RCP: peripapillary radial capillary plexus.

ROC curves for the identification of patients with DR (macular SCP, DCP) and glaucoma (peripapillary RCP) are illustrated in Fig 7. At the SCP level, ARI and manual algorithms performed better (p < 0.05) than all the other methods, but they did not significantly differ each other (p = 0.6). At the DCP level, the manual, Otsu, mean, and default algorithms had higher AUROCs than other methods, and the difference was significant (p <0.05) except for the pair mean and ARI (p = 0.1). At the ONH level, the fixed algorithm had the best performance to distinguish glaucomatous patients from healthy subjects, although the difference was significant only in comparison with multilevel (p = 0.029).

## Discussion

In the present study, we compared seven different methods to calculate vessel density on OCT-A angiograms in the macular and peripapillary areas in healthy subjects, and in patients with either DR or glaucoma. Methods tested included the ARI Zeiss proprietary algorithm v 0.6.1, a manual method, a static algorithm using the same fixed threshold for all eyes, three dynamic autothresholds (i.e., mean, default, Otsu) adapting their values in relationship to image properties, and a more complex algorithm employing a preprocessing filter followed by a multilevel thresholding strategy. We evaluated the inter-algorithm differences, reliability, and agreement. Moreover, we provided a calibration between algorithms to generate conversion formulas from one method to another. Finally, we investigated the ability of the algorithms to identify differences in vessel densities between healthy subject and patients with DR or glaucoma, and we compared their diagnostic performance.

Since its commercialization, OCT-A has gained increasing popularity in the ophthalmic community. The capability to image the retinal and choroidal vasculatures in non-invasive, fast, three-dimensional, depth-resolved fashion represents a considerable advantage of this technique over the traditional, dye-based diagnostic tests. Inspection of OCT-A angiograms may be of great help for the clinician in the diagnostic process. Identification of choroidal neovascularization in an asymptomatic patient with “dry” AMD or in a patient suffering from central serous chorioretinopathy are two examples where this new technology has a significant, clinical impact. OCT-A angiograms can be post-processed to obtain quantitative, objective measurements. Two established indices are the vessel density and, specific to the macular region, the fovea avascular zone (FAZ) area. The latter is a capillary-free zone corresponding to the foveola, while the former is the percentage of angiocube occupied by retinal vessels. A number of studies investigated changes in such indices in various ocular pathologies and their relationship with disease severity, activity, and response to treatments in several chorioretinal diseases, including diabetic retinopathy, [31, 32] retinal vein occlusion, [33] age-related macular degeneration, [11, 34] and retinal dystrophies. [13–15, 17] Vessel density applications are not limited to the retinal field, and are also useful in optic nerve diseases such as glaucoma [10, 22] and anterior ischemic optic neuropathy. [12] According to Cole et al., [4] vessel density could become a future surrogate endpoint for clinical trials. In a recent commentary, however, Garrity and Sarraf [35] claimed the need of additional technical and clinical research to fully elucidate properties and reliability of quantitative indices before their application in research trials and clinical practice. There is substantial evidence that the intra- and inter-operator repeatability of vessel density is good for images acquired in the same location, with the same angiocube size, machine, and quantification algorithm. [5, 6, 36] Nevertheless, some other factors both in the acquisition and in the post-processing phase can negatively affect its reliability. Some retinal diseases, such as cystoid macular edema, can profoundly disorganize the retinal architecture leading to inaccurate and unreliable results. [35] Low signal strength is correlated with lower vessel density, and this should be considered in patients with cataract or ocular surface disease. [5] Corvi et al [7] evaluated the reliability of quantitative indices, including vessel density, measured with seven different instruments in a cohort of healthy patients, and they found a poor reliability among the tested devices. These results are not surprisingly since different instruments have varying properties, including wavelength of laser beam, number of A-scans, algorithm used to detect flow, segmentation boundaries, and image resolution. In a previous study, we demonstrated that vessel density significantly differs across different angiocubes sizes since wider images are characterized by lower resolution compared to smaller, denser, scans. [9] Uji and colleagues [23, 37] evaluated the impact of multiple *en face* angiograms averaging, and found that it increases the image quality and impacts on the quantitative measurements reducing the variability. Only one study has provided some information about the impact of different post-processing imaging. Pedinielli et al [38] observed that the same image may lead to different macular vessel density values if quantified by means of skeletonization, mean thresholding, or proprietary AngioVue software (Optovue, Inc., Fremont, CA, USA). Differences between vessel density obtained by means of skeletonization and thresholding were predictable since they measure different units. Skeletonization does not take into account the vessel dimension and treats all the vessels in the same way irrespective to their size, so it minimizes the impact of large retinal vessels over the capillary network. On the contrary, image thresholding reveals the real percentage of retinal vasculature. Our results partially corroborate and expand the previous findings. Differences among methods were highly significant, and reliability and agreement values were mostly poor to moderate, with few exceptions. Differences were highest at the macular DCP with Manual, Default, Otsu, and fixed showing less differences, while ARI and Mean appear to report notably lower and higher values, respectively. The calibration equations revealed that systematic differences between algorithms were pretty consistent, and most of them exhibited a non-constant bias. A conversion formula was present for all the algorithms except for multilevel and fixed thresholds at the DCP. These two algorithms displayed very poor reliability and agreement, and, in the DCP, they surprisingly exhibited a negative correlation meaning that studies based on these algorithms may potentially lead to opposite results. On the other hand, we appreciated an excellent reliability and agreement between Otsu and default algorithms. These two methods demonstrated a small systemic bias for the macular area and, notably, no bias in the peripapillary area, suggesting that values derived from these algorithms are almost interchangeable. Conversion formulas provide a method to convert values from one algorithm to another, and, theoretically, longitudinal monitoring could be performed with images processed with different algorithms. However, most of the most methods exhibited a substantial, non-costant bias. Performance of these formulas should be validated in a different larger dataset before one may confidently switch from one algorithm to another during the follow-up. In the light of these considerations, we suggest to use the same algorithm in the longitudinal follow-up since processing methods are not directly interchangeable.

Several authors have conducted studies on large cohorts of healthy subjects to build normative databases for quantitative metrics, including vessel density. Coscas et al [39] were the first group to provide macular vessel density values in a Caucasian population. Iafe et al [40] also reported vessel density values in 70 healthy subjects, and Garrity et al [41] reported results of repeated analyses on the same cohort of patients updating the previous results based on more sophisticated software enabling projection removal and improvement of the segmentation algorithm with isolation of the intermediate capillary plexus. Bazvand et al [42] published a normative quantitative database for the papillary and peripapillary area. Other studies provided macular vessel density values in different populations (i.e., in Asia, Middle-East), and in pediatric subjects. [43–45] The results of these studies should be interpreted with extreme caution, and are not generalizable since vessel density values are dependent on the device, angiocube size, image averaging, and, as shown here, post-processing algorithm employed. Although not formally demonstrated in the present study, the updates of the proprietary software released by manufacturers could also cause changes in vessel density affecting longitudinal follow-up of patients.

Several studies unequivocally demonstrated that patients with DR and glaucoma have lower macular and peripapillary vessel density values, respectively. [2, 3, 21, 22, 24, 26, 31] In this study, we tried to replicate these well-established findings analyzing the same pool of images with seven different methods. All algorithms found a significant reduction in vessel density in the patients with glaucoma and DR compared to healthy subjects. We believe this is an important finding since it indicates that previous studies were not biased by the algorithm used at least for those tested in this study. We investigated which algorithm had the best diagnostic performance to discriminate patients from healthy subjects. No method outperformed the others in all the retinal plexuses, but the performance depended on the selected plexus. In the macular area, manual and ARI methods had the best AUROCs with regard to the SCP. At the DCP level, manual and autothresholds (i.e., mean, default, Otsu) algorithms had the best discriminating ability. As to the peripapillary RCP, all algorithms had a similar performance except for the multilevel, which had the smallest AUROC. These differences may be related to differences in the levels of grey in images segmented at different plexuses. Manual algorithms had a good discrimination ability in all the three analyzed plexuses, and this could indicate the flexibility of this method that allows the rater to manually adjust the threshold value based on a visual feedback. Unfortunately, manual algorithm is highly subjective and has a poor inter-rater reliability. Moreover, it is highly time-consuming and it requires trained raters.

Limitations of this study should be kept in mind. The retrospective nature dictated the fact that some variables (e.g., axial length) potentially affecting vessel density were not available. [46] Many methods to quantify vessel density have been published, and their relationship with those assessed in the present study remain unknown. Nevertheless, we tested a large number of methods using different strategies (including manual method, semiautomatic method with fixed threshold, semiautomatic methods with dynamic thresholds, and multilevel methods preceded by image filtering). All the images were acquired with the same device, and results might not be generalizable to other instruments. The calibration equations have not been tested on an external dataset, so they need to be fully validated. Also, they were based on the vessel density values of the study sample, and the relationship between two methods can differ for observations outside the range. Finally, our study was limited at the retinal vascular plexuses, and does not provide information about choroidal circulation.

In conclusion, we provide an extensive comparison of methods to quantify vessel density on OCT-A angiograms. Absolute values calculated with different algorithms are not directly interchangeable since methods have systemic differences, poor reliability, and poor agreement. Nevertheless, all the tested algorithms revealed significant differences between healthy and affected eyes, although they had different discriminatory abilities, which varied according to the plexus analyzed. This study indicates that longitudinal monitoring of the vessel density should be carried out with the same instrument, same scan pattern and location, and same algorithm. Studies adopting vessel density as an outcome should not rely on external normative database but include their own control groups. Knowledge of the properties for each algorithm could help researchers to select the best algorithm according to the plexus studied.

## Supporting information

S1 Dataset General dataset for demographic and clinical data. Readable table containing general data of the study population, including age, sex, eye laterality, presence of glaucoma, presence and stage of diabetic retinopathy, presence and type of diabetes, levels of hemoglobin A1c, presence of diabetic macular edema (DME), vertical cup-to-disc ratio (VCDR), retinal nerve fiber layer thickness (RNFL), and macular thickness. Missing or not applicable data are indicated as “NA”.
(CSV)

S2 Dataset Dataset for superficial capillary plexus. Optical coherence tomography angiography processed data for the macular superficial capillary plexus. DME: diabetic macular; OP1: operator 1; OP2: operator 2; OP3: operator 3. Missing or not applicable data are indicated as “NA”.
(CSV)

S3 Dataset Dataset for deep capillary plexus. Optical coherence tomography angiography processed data for the macular deep capillary plexus. DME: diabetic macular; OP1: operator 1; OP2: operator 2; OP3: operator 3. Missing or not applicable data are indicated as “NA”.
(CSV)

S4 Dataset Dataset for peripapillary radial capillary plexus. Optical coherence tomography angiography processed data for the peripapillary radial capillary plexus. DME: diabetic macular; OP1: operator 1; OP2: operator 2; OP3: operator 3. Missing or not applicable data are indicated as “NA”.
(CSV)
